# Biogenesis of Iron–Sulfur Clusters and Their Role in DNA Metabolism

**DOI:** 10.3389/fcell.2021.735678

**Published:** 2021-09-30

**Authors:** Ruifeng Shi, Wenya Hou, Zhao-Qi Wang, Xingzhi Xu

**Affiliations:** ^1^Shenzhen University-Friedrich Schiller Universität Jena Joint Ph.D. Program in Biomedical Sciences, Shenzhen University School of Medicine, Shenzhen, China; ^2^Guangdong Key Laboratory for Genome Stability and Disease Prevention and Marshall Laboratory of Biomedical Engineering, Shenzhen University School of Medicine, Shenzhen, China; ^3^Leibniz Institute on Aging—Fritz Lipmann Institute (FLI), Jena, Germany; ^4^Faculty of Biological Sciences, Friedrich-Schiller-University Jena, Jena, Germany

**Keywords:** iron-sulfur (Fe-S) clusters, genome stability, DNA replication, DNA repair, DNA metabolism

## Abstract

Iron–sulfur (Fe/S) clusters (ISCs) are redox-active protein cofactors that their synthesis, transfer, and insertion into target proteins require many components. Mitochondrial ISC assembly is the foundation of all cellular ISCs in eukaryotic cells. The mitochondrial ISC cooperates with the cytosolic Fe/S protein assembly (CIA) systems to accomplish the cytosolic and nuclear Fe/S clusters maturation. ISCs are needed for diverse cellular functions, including nitrogen fixation, oxidative phosphorylation, mitochondrial respiratory pathways, and ribosome assembly. Recent research advances have confirmed the existence of different ISCs in enzymes that regulate DNA metabolism, including helicases, nucleases, primases, DNA polymerases, and glycosylases. Here we outline the synthesis of mitochondrial, cytosolic and nuclear ISCs and highlight their functions in DNA metabolism.

## Introduction

Iron–sulfur (Fe/S) clusters (ISCs) are extremely ancient, small inorganic protein cofactors found in almost all organisms. Ferredoxin was discovered in the early 1960s, since then, the number of known Fe/S clusters-containing proteins has steadily increased. Until now, over 120 unique types of enzymes and proteins have been identified as ISC-containing proteins (Johnson et al., 2005). Until now, there are more than 200 known Fe/S proteins in human cells according to the UniProt database^1^. And bacteria contain a great variety of such proteins (Andreini et al., 2017). ISC proteins are found in the nucleus, cytosol, and mitochondria. The essentials of ISC proteins are reflected in the fact that they are required for many fundamental biochemical processes. For example, within mitochondria, the respiratory complexes I, II and III use many ISCs to transfer electrons which reduces ubiquinone by NADH or FADH, respectively. Within the nucleus, ISCs are functionally related to the maintenance of genome stability, RNA modification, and gene regulation. Specifically, ISCs are inserted into DNA repair enzymes, which fix DNA lesions according to the diffusing ability of an electron from an ISC along DNA (Arnold et al., 2016). Defects in mitochondrial ISC biogenesis can result in nuclear genomic instability (Veatch et al., 2009). Various nuclear DNA metabolic enzymes require ISCs to carry out DNA metabolism, including DNA primase, DNA polymerases (Klinge et al., 2007), DNA glycosylases (Alseth et al., 1999), and ATP-dependent DNA helicases (Rudolf et al., 2006; Gari et al., 2012; Stehling et al., 2012).

## Mitochondrial Iron–Sulfur (Fe/S) Cluster Biogenesis

There are three independent mechanisms that can synthesize ISCs in bacteria: the ISC assembly, methanoarchaeal sulfur mobilization (SUF) (Takahashi and Tokumoto, 2002), and nitrogen fixation (NIF) pathways (Mettert and Kiley, 2015). Each of these mechanisms shares the same steps: iron and sulfur ions are assembled at scaffold complexes. And then, the transfer system delivers the clusters to target proteins (Roche et al., 2013).

**FIGURE 1:**
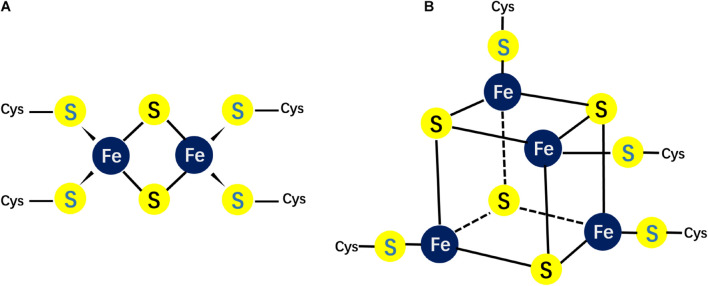
Different possible structures of Fe/S clusters. **(A)** The structure of the rhombic 2Fe–2S cluster; **(B)** the structure of the cubane 4Fe–4S cluster.

Eukaryotic mitochondria have one dedicated assembly pathway that inherits the ISC pathway from bacteria and integrate the NIF system components (Gisselberg et al., 2013; Roche et al., 2013). The ISCs in the cytoplasm and nucleus are assembled by the CIA pathway. In mammalian cells, there are two major forms of ISCs: 2Fe–2S and 4Fe–4S clusters (Figure 1). These two types of cofactors are generated by two related biochemical machineries in the cytosol (CIA pathway) and mitochondria (ISC pathway), respectively (Maio et al., 2020). The mitochondrial machinery assembly a necessary sulfur-containing intermediate that is exported to the cytoplasm and utilized for extramitochondrial ISCs assembly.

Mitochondrial ISC biogenesis has two functions: (1) to synthesize functional clusters in the mitochondria, and (2) to provide an essential precursor to the CIA pathway via the inner membrane exporter ABCB7 (Lill et al., 2015). Briefly, persulfide ions are generated by cysteine desulfurase (NFS1). Iron and sulfide ions are then delivered to a scaffold protein ISCU2 to form an initial 2Fe–2S cluster. Then chaperones transfer this 2Fe–2S cluster to a glutaredoxin, which subsequently delivers the 2Fe–2S cluster to the target protein or to the next Iron-sulfur assembly protein (ISA) complex. The ISA complex can condense two 2Fe–2S clusters into one 4Fe–4S center (Beilschmidt and Puccio, 2014). Major components involved in the ISC pathway are shown in Table 1. In sum, 2Fe-2S and 4Fe-4S proteins are made differently, with *de novo* 2Fe-2S clusters forming on the ISCU scaffold and 4Fe-4S clusters forming subsequently in a downstream step utilizing the ISA complex of proteins.

**TABLE 1:** Mitochondrial Fe/S cluster assembly components.

Complex	Human	Yeast	Function	Location
ISCU complex	ISCUs	Isu1/Isu2	Scaffold protein	Mitochondria, cytoplasm and nucleus
	NFS1	Nfs1	Cysteine desulfurase provides the sulfur	Mitochondria, cytoplasm and nucleus
	ISD11	Isd11	Stabilize binding partner of NFS1	Mitochondria and nucleus
	ACPM	Acp1	Bind to and stabilize Isd11	Mitochondria
	Ferredoxin	Yah1	Electron donor	Mitochondria
	Frataxin	Yfh1	Regulates NFS1 activity	Cytoplasm and nucleus
Cluster transfer complex	HSPA9	Ssq1	Co-chaperone of GLRX5	Mitochondria and nucleus
	HSC20	Jac1	Co-chaperone of GLRX5	Mitochondria, cytoplasm and nucleus
	GLRX5	Grx5	Transfers 2Fe–2S clusters to client proteins	Mitochondria
	ABCB7	Atm1	Exports Fe–S clusters from mitochondria	Mitochondria
	ALR	Erv1	Exports Fe–S clusters from mitochondria	Mitochondria and cytoplasm
ISA complex	ISCA1	Isa1	Assemble 4Fe–4S clusters	Mitochondria
	ISCA2	Isa2	Assemble 4Fe–4S clusters	Mitochondria
	IBA57	Iba57	Assemble 4Fe–4S clusters	Mitochondria
	NFU1	Nfu1	Transfer 4Fe–4S clusters to target proteins	Mitochondria

### 2Fe–2S Cluster Biogenesis

The 2Fe–2S clusters in rhombic form possess 2 sulfide ions and 2 irons, which coordinate four cysteinyl sulfhydryl side chains (Figure 1A). This rhombic-form cluster exhibits two oxidation states: the oxidized status with two Fe^3++^, and the reduced status with one Fe^3+^ and one Fe^2+^. As mentioned, the mitochondrial ISC pathway is essential for ISC biogenesis. This pathway starts with delivering iron and sulfur ions to scaffold protein ISCU2. ISCU has two isoforms: the mitochondrial isoform ISCU2 and cytosolic and nuclear isoform ISCU1. Cysteine desulfurase NFS1, which interacts with ISD11 and ACP to form a stable complex provides sulfur. However, the iron source of ISC is unknown yet. Frataxin (FXN) and ferredoxin2 (FDX2) are also important for ISCs *de novo* assembly. The former is thought to regulate NFS1 activity (Fox et al., 2019), while the latter is proposed to donate electrons for reduction (Cai et al., 2017; Gervason et al., 2019).

The eukaryotic cysteine desulfurase NFS1 is a pyridoxal phosphate-dependent enzyme. Sulfur is transferred from cysteine and activated into a persulfide form, which can be used for ISC assembly. In Cory et al. (2017), the first investigation into the eukaryotic NFS1 crystal structure revealed some key features: First, NFS1 binding to its substrate cysteine relies on a pyridoxal phosphate (PLP) cofactor. Second, there is a metal-binding cysteine site located in the C-terminus of NFS1. The activated sulfur abstracted is transferred to this cysteine site. Third, similar to the prokaryotic version of the enzyme, NFS1 forms a dimer. In the field of the enzymatic cycle, the PLP cofactor mediated the interaction of NFS1 and substrate cysteine. Then, NFS1 conformational change results in closing the activity site of cysteine with substrate and proceeding a nucleophilic attach (Johnson et al., 2005). During this second step, additional eukaryotic-specific subunits (ISD11 and ACP) interact with NFS1 and form a stable complex. ISD11 is a small protein of the LYR (Leu-Tyr-Arg motif) family, and ACP, which is an acyl carrier protein, regulates fatty acid synthesis. The long chain fatty acid of ACP is inserted into the helical center of the ISD11 subunit (Pandey et al., 2012; Parent et al., 2015; Cory et al., 2017). Finally, the persulfide sulfur is moved from NFS1 to the scaffold protein ISCU2 for ISC assembly.

The transfer of persulfur to the ISCU2 scaffold seems to be mediated by frataxin (Brancaccio et al., 2014; Fox et al., 2015). Frataxin is the earliest identified as a positive modulatory factor for NFS1 and ISC assembly. Frataxin interacts with NFS1 and enhance NFS1 cysteine desulfurase activity. Human neurodegenerative disease Friedreich’s ataxia results from the deficient of frataxin. Deletion of the yeast orthologous gene, Yfh1, leads to excess iron accumulation in mitochondria (Campuzano et al., 1996; Babcock et al., 1997; Karthikeyan et al., 2002). Because of frataxin’s weak Fe(II)-binding ability, it might be a potential iron donor to scaffold protein ISCU2 (Adamec et al., 2000; Yoon and Cowan, 2003). Structural studies have revealed that neither iron nor ISU oligomerization is essential for the interaction between bacterial frataxin, CyaY, and the ISU complex. Prokaryotic frataxin directly interacts with the bacterial desulfurase, IscS, but not the scaffold, IscU (Prischi et al., 2010). There are two apparently different reports for frataxin functions. One report suggested that frataxin promotes the interaction between NFS1 and substrate (Pandey et al., 2013), while frataxin was reported to promote sulfide transfer from NFS1 to ISCU1 (Parent et al., 2015). Furthermore, frataxin enhances sulfide transfer to ISCU1, forming a 2Fe–2S product on the ISCU scaffold (Bridwell-Rabb et al., 2014).

### Transfer of 2Fe–2S Clusters

Once the initial 2Fe–2S cluster has been generated by the ISC system in mitochondria, it is transferred to the glutaredoxin 5 (GLRX5) dimer with the help of HSC20 and HSPA9. This process requires energy, which is supplied by ATP hydrolysis carried out by HSPA9 (Dutkiewicz et al., 2003). The binding of the chaperones HSC20 and HSPA9 leads to the dissociation of the assembly complex consisting NFS1/ISD11/ISCU/ACP/FXN. Competition of FXN with HSPA9 for the LPPVK binding site on ISCU acts as a molecular switch between assembly and transfer complexes (Majewska et al., 2013; Manicki et al., 2014). Cluster–bound GLRX5 includes a 2Fe–2S cluster connecting a GLRX5 dimer. Each GLRX5 contributes one cysteine ligand to the 2Fe–2S cluster, while a second thiolate ligand coming from a GLRX5–bound glutathione stably binds GLRX5 (Banci et al., 2014). *glrx5* deletion in yeast cells has dysfunctional phenotypes in both mitochondrial ISC *de novo* biogenesis and cytosolic ISC assembly (Muhlenhoff et al., 2003; Uzarska et al., 2013).

### Mitochondrial 4Fe–4S Cluster Formation

The cubane-type cluster comprises 4 iron and 4 sulfide ions coordinated to four sulfhydryl side chains (Figure 1), which can be subdivided into low- or high-potential clusters^39^. The oxidation states of the low-potential clusters are the oxidized [2Fe^3+^, 2Fe^2+^] and the reduced [Fe^3+^, 3Fe^2+^] forms, while the oxidation states for the high potential clusters switch between the reduced [2Fe^3+^, 2Fe^2+^] and the oxidized [3Fe^3+^, Fe^2+^] forms. Hence, the two ferric-two ferrous state is shared by the two families. Within a cell, the most common clusters are 4Fe–4S clusters. The ISA system mediates the transformation of 2Fe–2S clusters into 4Fe–4S clusters in the mitochondrial matrix. Unlikely to mitochondrial ISC biogenesis system, the cytoplasm contains different machineries to assembly 4Fe–4S clusters. The biogenesis of cytoplasmic 4Fe–4S clusters rely on substrate exported from the matrix by ABCB7 (Lill et al., 2015).

In brief, ISCA1, ISCA2, and IBA57 are responsible for the generation of mitochondrial 4Fe–4S clusters. ISCA1 and ISCA2 interact with each other (Muhlenhoff et al., 2011; Beilschmidt et al., 2017), and structure study reveals that ISCA2, but not ISCA1, is able to bind IBA57 (Muhlenhoff et al., 2011; Beilschmidt et al., 2017; Gourdoupis et al., 2018). Two GLRX5-derived 2Fe–2S clusters are converted to a 4Fe–4S cluster on the ISCA1-ISCA2 complex. This process required the presence of IBA57 and the electron transfer chain NADPH-FDXR-FDX2 (Weiler et al., 2020). Finally, NFU1 promotes the 4Fe–4S cluster transfer from the ISCA1-ISCA2-IBA57 complex to apoproteins. However, one biochemical study indicated that only ISCA1, but neither ISCA2 nor IBA57, is needed for the maturation of the 4Fe–4S cluster in mouse skeletal muscle and in primary neurons (Beilschmidt et al., 2017).

ISA system is only responsible for mitochondrial 4Fe–4S clusters biogenesis. In the absence of the ISA complex, cells showed mitochondrial function defects with uncompromised cellular viability, since the sulfur-containing component required for cytoplasmic Fe/S biogenesis still can be exported from mitochondria.

## Biogenesis of Cytosolic and Nuclear Iron–Sulfur (Fe/S) Clusters

Except mitochondrial, there are also abundant ISC proteins located in cytosolic and nuclear in eukaryotic cells. And these proteins are involved in multiple biological processes. For example, DNA metabolism, iron regulation and metabolic catalysis. These processes are catalyzed by the CIA machinery. As discussed, the mitochondrial ISC assembly system generates an initial sulfur-containing intermediate and export the compound from mitochondria by the inner membrane ABC transporter, ABCB7 (Kispal et al., 1999; Gerber et al., 2004; Fosset et al., 2006; Lill et al., 2014). The exported intermediate is necessary for cytoplasmic ISC synthesis by the CIA system. However, chemical characterization and isolation of the intermediate is a subject of ongoing research. Glutathione (GSH) and the intermembrane space protein, ALR are important for this process (Kispal et al., 1999; Sipos et al., 2002; Pondarre et al., 2006; Cavadini et al., 2007). ALR is a FAD-dependent sulfhydryl oxidase. ALR inserts disulfide bridges into mitochondrial preproteins during their import into the intermembrane space (Mesecke et al., 2005). But another group reported that Cytosolic ISC protein maturation and iron regulation are independent of the mitochondrial Erv1/Mia40 import system. After the sulfur-containing compound was transferred to cytosolic, nine proteins of the CIA system are responsible for generating the cytosolic ISCs and inserting them into target proteins (Sharma et al., 2010; Basu et al., 2014; Netz et al., 2014). Major components involved in the CIA system are shown in Table 2. In the next section, the two essential steps of cytosolic and nuclear ISC assembly will be described.

**TABLE 2:** Cytosolic and nuclear Fe/S cluster assembly components.

Complex	Human	Yeast	Function	Location
CIA complex	CFD1	Cdf1	Scaffold protein	Cytoplasm
	NBP35	Nbp35	Scaffold protein	Cytoplasm and nucleus
	CIAPIN1	Dre2	Electron donor	Cytoplasm and nucleus
	NDOR1	Tah18	Electron donor	Cytoplasm
	IOP1	Nar1	Adaptor protein of CIA complex	Cytoplasm
	CIA1	Cia1	Transfer and insert Fe–S clusters into target proteins	Cytoplasm
	CIA2B	Cia2	Transfer and insert Fe–S clusters into target proteins	Cytoplasm and nucleus
	MMS19	Met18	Transfer and insert Fe–S clusters into target proteins	Cytoplasm and nucleus
	CIA2A	Absent	Specific maturation factor of IRP1	Cytoplasm

### Step 1 of Cytosolic and Nuclear Iron–Sulfur (Fe/S) Cluster Assembly

Similar to ISC biogenesis in mitochondria, the initial step of cytosolic and nuclear ISC synthesis is transient transfer a 4Fe–4S cluster to the cytosolic scaffold protein complex. This complex comprises two P-loop NTPases CFD1 and NBP35 (Roy et al., 2003; Hausmann et al., 2005; Netz et al., 2007; Stehling et al., 2018). CFD1 interact with NBP35 and form a heterotetrameric complex. This complex is able to coordinate two different types of 4Fe–4S clusters (Netz et al., 2012). One type of 4Fe–4S cluster can loosely bind to a conserved CX_2_C motif that is located at the C-termini of CFD1 and NBP35. The second type of 4Fe–4S clusters bind at a ferredoxin-like CX_1__3_CX_2_CX_5_C motif, which is located at the N terminus of NBP35. This motif is essential for NBP35 function. Interestingly, a pulse-chase experiment with ^55^Fe labeled yeast cells revealed the different labilities of the two ISCs associated with the CFD1–NBP35 complex (Pallesen et al., 2013). The 4Fe–4S cluster that binds to the N terminus of NBP35 is more stable than the 4Fe–4S cluster that binds the C-terminus, which transfers the loose-binding 4Fe–4S cluster to target proteins.

Another feature of cytosolic and nuclear ISC biogenesis that is similar to mitochondrial ISCs biogenesis is the dependency on a supply of electrons (Webert et al., 2014). The electron transfer chain of the CIA system comprises NADPH, NDOR1, and the Fe/S protein CIAPIN1 (Netz et al., 2010; Banci et al., 2013). NDOR1 is a key member of the electron transfer chain. It contains NADPH-, FAD- and FMN- binding domains. Protein-protein interaction and high-throughput studies have demonstrated that CIAPIN1 physically interacts with NDOR1 (Vernis et al., 2009). Dre2 is the CIAPIN1 yeast homolog: it is a crucial component of the cytosolic and nuclear ISC biogenesis system. The synthetically lethal effect was observed when deletion Dre2 and mitochondrial iron importers (Mrs3 and Mrs4) (Zhang et al., 2008). Dre2 contains a conserved C-terminal Fe/S domain which is responsible for coordinating one 2Fe–2S or one 4Fe–4S cluster by cysteine residues. The N-terminal of Dre2 is a SAM methyl-transferase-like domain, the middle linker domain of Dre2 mediate the connection of the N-terminus and C-terminus (Zhang et al., 2008; Netz et al., 2010, 2014).

### Step 2 of Cytosolic and Nuclear Iron–Sulfur (Fe/S) Cluster Assembly

The second step of cytosolic ISC biogenesis is initialed by releasing the newly assembled 4Fe–4S cluster from the CFD1-NBP35 scaffold complex. Then, the 4Fe–4S cluster is inserted into targeted proteins (Balk et al., 2004, 2005; Song and Lee, 2008, 2011). Both the CIA targeting complex and the iron-only hydrogenase-like protein, IOP1, are essential for this step reaction. Nar1 is the yeast ortholog of IOP1. Structure study of Nar1 revealed that the four conserved Cys residues, which are in the C-terminal of Nar1 are responsible for binding ISCs. The CIA targeting complex comprises CIA1, CIA2B, and human ortholog for the yeast methyl methanesulfonate-sensitivity protein 19 (MMS19). And these components physically interact with a large number of target proteins in the cytoplasm and nucleus (Srinivasan et al., 2007; Weerapana et al., 2010; van Wietmarschen et al., 2012; Stehling et al., 2013; Kassube and Thoma, 2020). The cytosolic ISC biogenesis contains two stages. In yeast, inactivation of early stage CIA assembles complex leads to the immaturity of Fe/S protein Nar1, while defect of late-stage proteins CIA1, CIA2B, and MMS19 do not affect ISC insert to target proteins (Balk et al., 2004). Base on this study, the early and late stage of CIA system is connected by Nar1via an unknown mode of action (Stehling et al., 2013).

CIA targeting complex component CIA1 contains seven WD40-repeat domains. The structural analysis demonstrated that these seven WD40-repeats distribute around a central axis, which functions as binding region docking site of the CIA targeting complex (Srinivasan et al., 2007). Point mutation of CIA1 has revealed that the conserved, surface-exposed residue R127 is responsible for assembling other subunits of cytosolic Fe/S protein (Paul and Lill, 2015). The conserved Cys residue is important for CIA2 function, which is also conserved in eukaryotes (Weerapana et al., 2010; Luo et al., 2012; Stehling et al., 2013). Knockout human CIA2B or its ortholog Cia2 suppresses the Fe/S proteins maturation (Chen et al., 2012). MMS19 contains 4 HEAT repeats at N-terminal. As the largest component of CIA, MMS19 is associated with the multitude of biological processes. For example, impaired chromosome segregation, defective double-strand break repair via homologous recombination, and immature cytosolic and nuclear Fe/S proteins (Prakash and Prakash, 1977; Lauder et al., 1996; Kou et al., 2008; Ito et al., 2010). For a long time, it was difficult to associate these phenotypes of MMS19-deficient cells with one molecular function. Until known that MMS19 is involved in cytosolic ISC biogenesis, this problem was resolved (Gari et al., 2012; Stehling et al., 2012). As a major determinant of the CIA targeting complex, MMS19 interacts with numerous target proteins and promotes the insertion of ISCs into them, including key enzymes in DNA synthesis (POLD1, PRIM2), DNA repair [XPD, DNA2 (DNA replication helicase/nuclease 2)], and telomere length regulation (RTEL1). Deletion of these enzymes, respectively, phenocopied variant MMS19 depletion defects.

## The Close Link Between Iron–Sulfur (Fe/S) Clusters and Genome Integrity

Mitochondria are organelles with a double-layer membrane found in most eukaryotic organisms. They generate most of the cellular chemical energy via oxidative phosphorylation. In addition to supplying energy, mitochondria are also involved in multitude of cellular biochemical processes such as programmed cell death, reactive oxygen species (ROS) production, and ISCs biogenesis. Biochemical studies revealed that mitochondrial DNA (mtDNA) defection result in nuclear genome instability and reduction of cells’ viability in yeast. This effect is due to the important role of mitochondria ISC biogenesis (Veatch et al., 2009). As mentioned, down-regulation of Nar1 is sufficient to alter nuclear genome stability (Gari et al., 2012). Consistent with this study, deletion of Zim17, an important component of ISC biogenesis, leading to genomic instability (Diaz de la Loza Mdel et al., 2011; Stehling et al., 2013). Given that many enzymes that are required for DNA synthesis and repair harbor ISC cofactors, these observations suggest that defects in Fe/S biogenesis and distribution are likely to be the origin of genomic instability (Ben-Aroya et al., 2008; Veatch et al., 2009; Gari et al., 2012; Stehling et al., 2012; Stehling et al., 2013). For example, MMS19 was identified as a gene involved in transcription conducted by RNA polymerase II, nucleotide excision repair (NER), and methionine biosynthesis (Prakash and Prakash, 1977; Thomas et al., 1992). In yeast, the essential transcription factor IIH (TFIIH) complex is required for transcription-coupled NER (Lauder et al., 1996). MMS19 is not a component of the TFIIH complex, while numerous studies demonstrate that MMS19 is crucial to maintain the cellular Rad3 (XPD in human) protein level, a component of the TFIIH (Kou et al., 2008). Consistent with these findings, human MMS19 homolog also is reported to be involved in the NER pathway by regulating TFIIH function. In addition to regulating TFIIH function, MMS19 also directly interacts with CIA components CIA1 and CIA2B. And this interaction is important for regulating chromosome segregation and telomere length (Askree et al., 2004; Ito et al., 2010). All these MMS19 functional studies reveal the different phenotypes observed in MMS19 deficient cells (Gari et al., 2012; Stehling et al., 2012).

The function of MMS19 in DNA metabolism has been reported in multitude of ways. Many biochemical studies demonstrate that MMS19 and other CIA complex components directly interact with diverse DNA metabolism enzymes, such as DNA helicases [XPD, FANCJ (Fanconi anemia complementation group J)], and RTEL1, DNA polymerase subunits (POLD1, POLA1, and POLE1), the nuclease DNA2, the DNA glycosylase NTHL1, and the DNA primase PRI2. With the help of MMS19, these enzymes coordinate an ISC. Biochemical studies have revealed that MMS19 mediates the interaction of XPD and TFIIH, which is important for DNA metabolism. In yeast, deletion of Met18/Mms19, CIA complex components, increases phosphorylation of Rad3 and promotes Rad3-dependent gene expression (Gari et al., 2012; Stehling et al., 2012). Consist with this finding, cells lacking CIA complex proteins are very sensitive to DNA damage events, e.g., UV and chemical agents. Based on these studies, MMS19 not only is involved in the CIA complex for the maturity of target ISC proteins, but also plays a crucial role in DNA metabolism.

Recently, one biochemical study also indicated that inhibition of ISCs synthesis via NFS1 depletion in elevated O2 environment led to decreased POLE protein level. This perturbation reduces Pol ε activity and causes replication stress (Sviderskiy et al., 2020).

## Iron–Sulfur (Fe/S) Clusters and DNA Replication

High-fidelity DNA replication ensures the accurate transmission of parental genetic information to daughter cells. This process is coordinated by numerous enzymes (Bell and Dutta, 2002). Firstly, the DNA helicases open the double-stranded DNA. Then, the DNA primases initiate DNA synthesis via assembling short RNA primers, which are extended by DNA polymerases. DNA polymerases then utilize the two parental DNA strands as templates to synthesize complementary strands, but not to start *de novo* DNA replication (Bell and Dutta, 2002). During the DNA replication process, DNA2, a helicase/nuclease, is critical for lagging strand DNA replication via processing Okazaki fragment (Kang et al., 2010). The ISCs are critical for the proper functions of all three types of enzymes (Table 3). In the next section, we will describe the three types of replication factors that coordinate these crucial ISCs.

**TABLE 3:** DNA metabolism enzymes with Fe/S clusters.

Human	Yeast	Function	Associated disease
PRIM2	Pri2	Subunit of DNA primase, DNA synthesis and double-strand break repair	–
CHLR1	Chl1	Helicase, sister chromatid cohesion, heterochromatin organization	Warsaw breakage syndrome
DNA2	Dna2	Helicase/nuclease, DNA repair	–
FANCJ	Absent	Helicase	Fanconi anemia
RTEL1	Absent	Helicase	Hoyeraal–Hreidarsson syndrome
XPD	Rad3	Helicase	Xeroderma pigmentosum, Cockayne syndrome
POLA	Pol1	Catalytic subunit of polymerase α,	–
POLD1	Pol3	Catalytic subunit of polymerase δ,	–
POLE1	Pol2	Catalytic subunit of polymerase ε,	–
MUTYH	Absent	DNA glycosylase	–
NTHL1	Ntg2	DNA glycosylase	–

### Iron–Sulfur (Fe/S) Clusters and Helicases

Helicases are a class of motor proteins that can unwind structured nucleic acids in an ATP-dependent manner. In this way, helicases can regulate many different processes that depend on strand separation during DNA metabolism (Lohman and Bjornson, 1996), including transcription, DNA replication, DNA repair, and telomere length regulation. Thus, helicases are important for genomic stability (Patel and Donmez, 2006; Brosh and Bohr, 2007; Lohman et al., 2008; Pyle, 2008). Helicases are classified into six super-families according to their primary amino acid sequences, and ISCs exist in numerous helicases.

In the helicase super-family 1, DNA2 is a multifunction enzyme not only involved in DNA replication, but also in double-strand DNA break (DSB) repair and telomere maintenance (Budd et al., 2005; Kang et al., 2010; Balakrishnan and Bambara, 2011). AddAB which contains a 4Fe–4S cluster, is a helicase-nuclease complex in bacteria. Eukaryotic helicase-nuclease DNA2 putative metal-binding motif was identified by sequence alignment with AddAB. Interestingly, structure studies revealed that four conserved Cys residues which are coordinated ISCs exist in the nuclease domain. This suggests that ISCs might function in stabilizing the nuclease domain conformation (Yeeles et al., 2009). Biochemical studies confirmed that yeast DNA2 coordinates ISCs by its conserved Cys residues (Pokharel and Campbell, 2012). ISC binding cysteine residues mutation results in nuclease activity and ATPase function defects in DNA2. However, the DNA binding ability of DNA2 is normal. Another biochemical study revealed that pro residue at position 504 of DNA2 is crucial to stabilize the ISC. These studies confirmed that the ISC regulates DNA2 nuclease and helicase activities by mediating conformational changes.

In the helicase super-family 2, an XPD homolog from Archaea was the first DNA repair helicase to be identified. A sequence alignment revealed that all the XPD helicase family members contain four highly conserved Cys residues. These conserved Cys residues which coordinate an ISC are crucial for 5′–3′ DNA helicases activity. The XPD helicase family comprises XPD and several related super-family 2 DNA helicases including DDX11/ChlR1 (DEAD/DEAH box helicase 11), RTEL1 (regulator of telomere elongation 1), and FANCJ (Fanconi anemia complementation group J). Many human diseases are linked to mutations in these three proteins (Table 3; White, 2009; Wu and Brosh, 2012).

XPD is a crucial subunit of the transcription initiation factor TFIIH, which is involved in NER and transcription (Compe and Egly, 2012). TFIIH comprises two major functional subcomplexes, a core complex (XPB, p8, p34, p44, p52, and p62), and a CAK (CDK–activating kinase) complex (cyclin H, CDK7, and MAT1). Helicase XPD is an important bridge between these two subcomplexes.

The mutations of XPD gene are related to three genetic diseases: xeroderma pigmentosum (XP), Cockayne syndrome (CS), and trichothiodystrophy (TTD) (White, 2009; Wu and Brosh, 2012). All three disorders have similar characteristics, with patients’ skin being hypersensitive to sun exposure. This is due to the defect of the NER pathway (Lehmann, 2003). In 2006, biochemical and spectroscopic analyses indicated that XPD coordinates a 4Fe–4S cluster, which is a key determinant of XPD helicase activity. This finding significantly contributes to revealing the molecular differences of how mutations in a single gene result in different diseases (Stehling et al., 2012). Subsequently, structural analysis showed that the 4Fe–4S domain forms a channel with an arch domain, that can accommodate single-stranded DNA (ssDNA) (Fan et al., 2008; Liu et al., 2008; Wolski et al., 2008). Mutational analysis of conserved cysteine residues in the Fe/S domain of XPD indicated that an intact Fe/S domain is essential for helicase activity and/or stabilizing the protein structure (Liu et al., 2008; Pugh et al., 2008). For patients with XP, mutations in XPD primarily inhibit helicase activity without affecting the protein structure. Interestingly, all XP-causing mutations are conserved in archaeal XPD (Fan et al., 2008; Liu et al., 2008). However, most of the mutated residues in TTD are not conserved in the archaeal protein (Liu et al., 2008). In TTD patients, R112H exchange is the most common mutation. This amino acid substitution leads to loss of XPD helicase activity and deficiency of NER (Dubaele et al., 2003). Biochemical and structural studies demonstrated that this Arg residue is essential for the Fe/S domain. These findings underscore the structural role played by the ISC in helicase activity and highlight the close relationship between ISCs and DNA replication. In addition to these disease-associated mutations, other mutations of XPD destabilize the helicase structure and compromise interactions between the two TFIIH sub-complexes (Dubaele et al., 2003; Liu et al., 2008).

FANCJ, which was able to interact with the breast cancer C-terminal (BRCT) repeats of BRCA1, is another important member of the XPD helicase family (Cantor et al., 2001). FANCJ has been identified as the gene that is mutated in the J complementation group of Fanconi anemia (FA), a genome instability disorder with an elevated risk of developing cancer. FANCJ is known as an anti-oncogene because of its functions in DNA repair (Wu and Brosh, 2009). The substitution A349P in FANCJ is a common mutation seen in patients with FA (Wu et al., 2010). Although this alanine is not a conserved site in the XPD helicase family, the residue is near the fourth highly conserved cysteine residue in the ISC. Consistent with this, recombinant FANCJ-A349P protein was shown to decrease iron content and inhibit the separation of double-stranded DNA (dsDNA) (Wu et al., 2010). This finding indicates that, like XPD helicase, the catalytic activities of FANCJ critically rely on an intact Fe/S domain.

DDX11/CHLR is the third member of the XPD helicase family. The genetic disease Warsaw breakage syndrome (WABS) arises from a mutation in the human CHLR1 gene (van der Lelij et al., 2010). In *S. cerevisiae*, a mutation in *chl1* causes chromosome loss and unusual mating phenotypes (Liras et al., 1978). Consistent with this finding, mutations in *chl1* or CHLR1, the human homolog, show similar results (Skibbens, 2004; Parish et al., 2006). Unsurprisingly, patient-derived mutations also abolish helicase activity due to their perturbance of DNA binding and DNA-dependent ATPase activity (Wu et al., 2012).

### Iron–Sulfur (Fe/S) Clusters and DNA Primase

A common feature of all DNA polymerases is that they are unable to initiate *de novo* synthesis of a DNA strand; they can only elongate an existing strand. Synthesis of a new strand can only begin from a primer with the 3′-OH end. Hence, a primase is required to catalyze the priming, form a primer, and initiate DNA replication. Primase in eukaryotic cells comprises two subunits, the catalytic PRIM1 subunit, and a large subunit PRIM2, both interacted with DNA polymerase-α (Frick and Richardson, 2001; Kang et al., 2010). Although only the PRIM1 subunit possesses catalytic activity, PRIM2 is also crucial for primase function (Zerbe and Kuchta, 2002). Spectroscopic analysis indicates that PRIM2 is able to bind a 4Fe–4S cluster, which is conserved from Archaea to eukaryotic cells (Weiner et al., 2007). Without this ISC, its enzymatic activity is compromised. High-resolution structural studies show that the conserved Lys314 in the C-terminal domain of human PRIM2 is supported by the 4Fe–4S cluster. This Lys314 mutant abolishes primer synthesis and DNA binding (Vaithiyalingam et al., 2010). This finding suggests that ISCs facilitate DNA binding via organizing the protein surface (Vaithiyalingam et al., 2010).

In addition, ISCs serve as a major determinant for regulation through their physical interactions with other proteins involved in DNA replication, the DNA damage response, stalled replication fork, and telomere maintenance (Weiner et al., 2007). The N- and C-terminal domains of PRIM2 folded together and are connected by a flexible 18-residue linker (Baranovskiy et al., 2018). Crystal structure studies reviewed that there are three metal-binding sites in the DNA primase, a Zn^2+^-binding site, a PRIM1 catalytic site which coordinates two Mg^2+^ (or Mn^2+^) ions, and a 4Fe–4S binding site in PRIM2. Furthermore, PRIM2 has four conserved Cys residues: Cys287, Cys367, Cys384, and Cys424, which are important for coordinating ISC. Point mutation of these Cys residues cause instability of both PRIM1 and PRIM2. The unstable structure of PRIM1 and PRIM2 lead to dysfunction of DNA polymerase-α primase complex and stalled replication fork (Liu and Huang, 2015). In fact, even a single point mutation of the conserved Cys residues is sufficient to reduce the activities of DNA primase and DNA polymerase. This result indicates that ISCs have an important role in enzyme functions^(12)^.

### Iron–Sulfur (Fe/S) Clusters and Polymerases

In eukaryotes, four types of class B family DNA polymerase complexes mediate replication and replication-associated genome maintenance. During normal replication, DNA polymerases (Pol) α, δ, and ε are responsible for replication fork extension. While the fourth polymerase, Polζ, is required for DNA synthesis at damaged sites (Johansson and Macneill, 2010). These polymerases are comprised of catalytic, regulatory, and accessory subunits (Burgers et al., 2001). Biochemical and structural studies demonstrate that there are two metal-binding motifs with conserved cysteine (CysA and CysB) located at Pol α, Pol δ, and Pol ε C-terminal catalytic subunits. At first, it was reported that these two metal-binding motifs coordinate Zn^2+^ ions (Evanics et al., 2003; Klinge et al., 2009). And they are essential for the stability of replisome. However, synthetically lethal effects are observed in yeast containing a single point mutant in the Pol3 CysB motif with essential components (DRE2, NBP35, and TAH18) of CIA complex (Chanet and Heude, 2003). Furthermore, pulse-chase ^55^Fe experiment, UV–Vis, and electron paramagnetic resonance (EPR) spectroscopic studies proved that the CysB motifs of all B-family DNA polymerases coordinate ISCs rather than Zn^2+^ (Netz et al., 2011; Suwa et al., 2015; Ter Beek et al., 2019). Overexpression *S. cerevisiae* Polδ subunit Pol31 enhances the ability of binding ISCs (Sanchez Garcia et al., 2004). Consist with this study, Polζ catalytic subunit Rev3 also coordinates the 4Fe–4S cluster in CysB. And the 4Fe–4S cluster is crucial for stabilizing the polymerase complex (Baranovskiy et al., 2018). Together, these findings suggest that the proper activities of DNA polymerases require 4Fe–4S cluster coordination. In addition to CysB, CysA is also important for the interaction between PCNA with Polδ on DNA. PCNA is a major determinant for regulating DNA replication and cell cycle. Notably, the DNA polymerase and exonuclease of Polδ were regulated by coordinated ISC (Jozwiakowski et al., 2019). In addition to class B-family polymerase complex, biochemical studies revealed that D-family polymerases also coordinate ISCs in their CysB motif. Furthermore, ISCs are important for polymerase complex formation. Point mutation of conserved Cys residue in Pol3 results in the reduction of coordinated ISC and disassociation with Polδ subunits Pol31 and Pol32. Moreover, Pol3 and the Fe/S biosynthetic genes are synthetic lethal, indicate that ISC is an essential cofactor for DNA polymerase to regulate its structure and functions (Chanet and Heude, 2003).

## Iron–Sulfur (Fe/S) Clusters Protein and DNA Repair

Oxidation, deamination, and alkylation are likely to induce single base damage in DNA. Base excision repair (BER) is a highly conserved cellular biochemical process that repairs damaged bases throughout the cell cycle (Krokan and Bjoras, 2013). BER is started from DNA glycosylases, which recognize and remove damaged or inappropriate bases by forming AP sites. Then, these AP sites are cleaved by an AP endonuclease. Finally, according to the length of the resulting single-strand break, the damaged DNA can be repaired by short-patch or long-patch BER (Wallace, 2013). During this process, many DNA glycosylases contain Fe/S cofactor (Guan et al., 1998; Alseth et al., 1999; Hinks et al., 2002). The *E. coli* endonuclease III (Endo III) is the first known DNA glycosylase that coordinates a 4Fe–4S cluster. The interaction of Endo III with the DNA phosphate backbone is dependent on its ISC (Kuo et al., 1992). The *E. coli* MutY is an adenine DNA glycosylase involved in BER. Structurally like Endo III, MutY coordinates a 4Fe–4S cluster (Guan et al., 1998), which is important for MutY structure stability and recognition of substrates (Porello et al., 1998; Lu and Wright, 2003). Electrochemical studies showed that DNA binding of Endo III and MutY shifts the redox potentials of the 4Fe–4S clusters, which sense DNA lesions via electron transfer (Boal et al., 2005). Consist with MutY, the mammalian homolog MUTYH also functions in fixing oxidation caused DNA lesions (McGoldrick et al., 1995).

To date, there are no reports to suggest that DNA topoisomerase or ligase is coordinated with the ISC. However, both have been linked to cellular ISCs metabolism. Eukaryotic DNA topoisomerase II (Topo II) is able to modulate negative supercoiling DNA in an ATP-dependent manner. The inhibition of Topo II leads to a loss of chromosomal supercoiling and furthermore results in the upregulation of oxidative phosphorylation (Dahan-Grobgeld et al., 1998), which increases ROS levels (Nosal et al., 2014). The inhibition of Topo II induces the DNA damage response, upregulation of iron uptake, and ISC biosynthesis (Dwyer et al., 2007).

## Concluding Remarks and Future Perspectives

Much research conducted over the past decade has greatly advanced our understanding of how the ISCs assemble and insert into target proteins in mitochondria, cytoplasm, and nucleus. However, there is still much to learn. For example, most of the proteins functioning in these pathways have been identified, but a complete picture of how ISC formation is regulated remains unclear. Recently, one group reported that acylated ACP1 may regulate ISCs *de novo* assembly via its dynamic interaction with ISD11. Upon high acetyl-CoA, mtFAS promotes long fatty acyl chain synthesis and acylated ACP1 binds to NFS1-ISD11. The long fatty acyl chain is able to stabilize the NFS1-ISD11-ACP1 complex and promote ISCs *de novo* assembly. On the contrary, cells that lack acylated ACP1 exhibit lower efficiency of ISCs assembly (Van Vranken et al., 2018). It is undeniable, however, that the regulation of ISC formation is crucial for cell survival.

ISC formation is controlled by several comprehensive mechanisms, including that (1) the Fe/S machinery requires delicate allosteric control, (2) the ISC delivery variations are regulated by carrier proteins, and (3) the expression levels of the Fe/S assembly protein are transcriptionally regulated. Despite great progress has been made, more research is needed to gain further insights into these processes.

Recent studies using mass spectrometry have identified many phosphorylation sites of NFS1; it has also been shown that mitochondria contribute to NFS1’s phosphorylation, which is required for its activity. Since lacking sulfur from cysteine stops the ISC synthesis, this Nfs1 phosphorylation in mitochondria has the great potential to regulate the entire ISC assembly process (Rocha et al., 2018). However, although the crystal structure of the human NFS1/ISD11/ACP complex has been observed, the phosphorylated residues were not detected and remain unclear (Cory et al., 2017).

Similarly, in the cytoplasm of mammalian cells, ISCU is phosphorylated by mTORC1. This phosphorylation event enhances the stability of the protein and promotes ISC assembly (La et al., 2013).

The degradation mechanism of the ISC is also unclear. One unique feature of 4Fe–4S is that it can be cleaved to either one 3Fe–4S cluster or two 2Fe–2S clusters. For instance, the 4Fe–4S cluster in a nitrogenase Fe-protein can be converted into two 2Fe–2S clusters (Sen et al., 2004). ISCs can also serve as sulfur donors for other sulfur-containing protein cofactors, such as biotin and lipoic acid, in a self-sacrificing fashion. Exploring the mechanism of this cleavage is essential to understanding the function of these proteins. We believe that a combination of the developing approaches in the structural, biochemical, and cell biological fields will deepen our knowledge of the molecular mechanisms of assembly, insertion, and regulation of the ISCs in target proteins.

A steadily increasing number of ISC proteins that function in genome integrity maintenance have been identified. Thus, the next major research challenge is elucidating their molecular mechanisms. To date, most of the studies support that the ISC stabilizes the structure of DNA metabolism proteins (White and Dillingham, 2012). Besides that, Barton’s group found that electron transport happens over a long distance of DNA. DNA lesions disrupt this charge transfer, which changes the redox-active status of the ISC in DNA. This proposes that, ISC is the key of how DNA glycosylases distinguish the intact and damaged bases. Similarly, primer synthesis by primase also requires the 4Fe–4S cluster although the underlying mechanisms remain unclear. One DNA-mediated electrochemistry experiment demonstrated that a reversible on/off switch in DNA primase for DNA binding is the oxidation state of the 4Fe–4S cluster. Moreover, primer synthesis is regulated by both the conserved charge transfer pathway through primase and DNA charge transport chemistry. This finding suggests that the primase uses DNA charge transport for redox signaling of 4Fe–4S clusters thus provides a chemical basis for understanding the precise regulation of primase activity and supports the notion of a fundamentally new redox switch model for substrate handoff (O’Brien et al., 2017).

Overall, the ISCs coordinate with key proteins in DNA metabolism. This coordination with the ISC regulates the target proteins via (1) stabilizing their structures, (2) mediating their local conformational changes, and (3) facilitating DNA charge transport. Improving our understanding of the critical roles played by ISCs in DNA replication and repair enzymes will ultimately help us solve the great mysteries around the DNA metabolism enzymes critical to life.

## Author Contributions

RS and XX conceived the scope and schemes of this review manuscript. RS wrote the first draft. WH, Z-QW, and XX revised and finalized the manuscript. All authors read and approved the submitted version.

## Conflict of Interest

The authors declare that the research was conducted in the absence of any commercial or financial relationships that could be construed as a potential conflict of interest.

## Publisher’s Note

All claims expressed in this article are solely those of the authors and do not necessarily represent those of their affiliated organizations, or those of the publisher, the editors and the reviewers. Any product that may be evaluated in this article, or claim that may be made by its manufacturer, is not guaranteed or endorsed by the publisher.

## Footnotes

1
https://www.uniprot.org/
